# Propensity Matched Analysis of 14 French Suture‐ Versus Plug‐Based Vascular Closure During Transfemoral Transcatheter Aortic Valve Replacement (TAVR)

**DOI:** 10.1002/ccd.31626

**Published:** 2025-05-28

**Authors:** Tobias Lerchner, Lars Michel, Klaus Tiroch, Tienush Rassaf, Markus Krane, Marc Michael Vorpahl, Hendrik Ruge

**Affiliations:** ^1^ Department of Cardiology and Vascular Medicine, West German Heart and Vascular Center University Hospital Essen Essen Germany; ^2^ Department of Cardiology Heart Center Bodensee Konstanz Germany; ^3^ Department of Cardiovascular Surgery, Institute Insure, German Heart Center Munich, School of Medicine & Health Technical University of Munich Munich Germany; ^4^ Department of Surgery, Division of Cardiac Surgery Yale School of Medicine New Haven Connecticut USA; ^5^ DZHK (German Center for Cardiovascular Research)—Partner Site Munich Heart Alliance Munich Germany; ^6^ Department of Cardiology, Rhythmology and Vascular Medicine Helios Clinic Siegburg Siegburg Germany

**Keywords:** access site complications, low‐profile sheath, Pb‐VCD, plug‐based vascular closure, Sb‐VCD, structural heart, suture‐based vascular closure, TAVR, transfemoral

## Abstract

**Background:**

Plug‐based vascular closure devices (Pb‐VCD) and suture‐based vascular closure devices (Sb‐VCD) are used for percutaneous vascular access site closure during transcatheter aortic valve replacement (TAVR). Until now, no clear superiority of either device was shown in studies comparing 18 F VCDs solely. However, there is no data exclusively comparing the 14 F Pb‐VCDs against Sb‐VCDs after novel 14 F low‐profile third‐generation heart valve delivery sheath use with focus on vascular complications.

**Aims:**

This study aimed to compare the safety and efficacy of 14 F Pb‐VCD to Sb‐VCD following 14 F low‐profile transcatheter heart valve delivery sheath use during TAVR.

**Methods:**

We performed a retrospective, propensity score‐matched comparison of patients receiving either the 14 F Pb‐VCD or the Sb‐VCD after 14 F low‐profile third‐generation heart valve delivery sheath use during TAVR. Valve academic research consortium‐3 (VARC‐3) criteria were used to define the primary endpoint of major and minor vascular complications at the access site. Secondary endpoints included length of hospital stay and in‐hospital mortality.

**Results:**

Two hundred and fifteen (Sb‐VCD) and 169 (Pb‐VCD) patients were included in propensity score matching and resulted in 69 matched patient pairs. The primary endpoint of major vascular complications was comparable between the groups (8.7% [Sb‐VCD] vs. 5.8% [Pb‐VCD], *p* = 0.511), whereas minor vascular complications were more frequent in the Pb‐VCD group (2.9% vs. 11.6%, *p* = 0.049). Secondary endpoints of length of hospital stay (*p* = 0.270) and in‐hospital mortality (*p* = 0.366) were balanced between the groups.

**Conclusion:**

14 F Pb‐VCDs are associated with significantly higher rates of VARC‐3 defined minor vascular complications after 14 F delivery sheath utilization during TAVR, not leading to increased in‐hospital patients' mortality. Adequate vascular closure following transfemoral TAVR remains of high clinical significance and continuous efforts are needed to optimize vascular access and closure strategies.

**Abbreviations**
BMI: body mass index
CFA: common femoral artery
F: French
NOAC: new oral anticoagulants
NYHA: New York Heart Association
Pb‐VCD: plug‐based vascular closure device
Sb‐VCD: suture‐based vascular closure device
TAVR: transcatheter aortic valve replacement
TF‐TAVR: transfemoral‐transcatheter aortic valve replacement
THV: transcatheter heart valve
VARC‐2: valve academic research consortium‐2
VARC‐3: valve academic research consortium‐3
VCD: vascular closure device

## 1 Introduction

Transfemoral aortic valve replacement (TAVR) has revolutionized the treatment of highly symptomatic patients with aortic stenosis. Despite the rapid development of novel therapeutic options for favorable procedural outcomes during transfemoral TAVR, rates of vascular adverse events remain high with significant impact on morbidity and mortality in this highly vulnerable patient cohort [1, 2, 3]. Here, Valve Academic Research Consortium‐3 (VARC‐3) defined bleeding events and arterial obstructions like dissections or stenosis are the most frequent vascular complications observed [4, 5].

Large bore vascular closure techniques are extensively studied since early TAVR days, initially focussing on the suture‐based Perclose ProGlide (Abbott Vascular, Illinois, USA) technique for percutaneous closure during TAVR. Recently, the MANTA (Teleflex Inc, Morrisville, USA) plug‐based vascular closure device (Pb‐VCD) was compared against the suture‐based vascular closure device (Sb‐VCD) [4]. Current evidence suggests that no clear superiority of neither system exists, but physicians' choice could be determined individually by patients' phenotype. In addition, most studies conducted included only 18 F VCDs or analyzed 14‐ and 18 F devices together and had outdated endpoints like the Valve Academic Research Consortium‐2 (VARC‐2) criteria. However, results of randomized trials hypothesize that 14 F VCD size rather than device type could lead to decreased rates of vascular complications [5].

In tandem with advancements in the development of VCDs for closure of the large‐bore puncture site, third‐generation transcatheter heart valves (THV) were designed to lower vascular trauma by smaller sheath profiles and expandable or integrated delivery sheath concepts [6]. Recently, integrated delivery sheaths, such as the Medtronic EnVeo PRO low‐profile system, showed comparable results when tested against an expandable sheath concept [2]. Despite the small diameter (14−16 French) of the integrated delivery sheaths, the need for pre‐ and postdilatation requiring large bore sheath exchanges with multiple vessel entries is discussed as a source of vascular trauma. However, other low‐profile delivery sheaths without the need for pre‐ and postdilatation or exchange with multi‐vessel entries, such as the Medtronic Sentrant, exist. While both third‐generation Medtronic systems, either implanted using EnVeoPro or Sentrant, are of low profile and utilized in routine clinical practice, both are different in the handling, and the need for sheath exchange potentially contributes to the risk of vascular trauma.

Thus, the aim of the present study was to compare the safety and efficacy of the 14 F MANTA (Teleflex Inc, Morrisville, USA) Pb‐VCD to the Perclose ProGlide (Abbott Vascular, Illinois, USA) Sb‐VCD following 14 F low‐profile THV delivery sheath use during TAVR and to compare our findings to previous described rates of vascular complications after larger 18 F VCD use (Central illustrations 1).

**Central Illustration 1:**
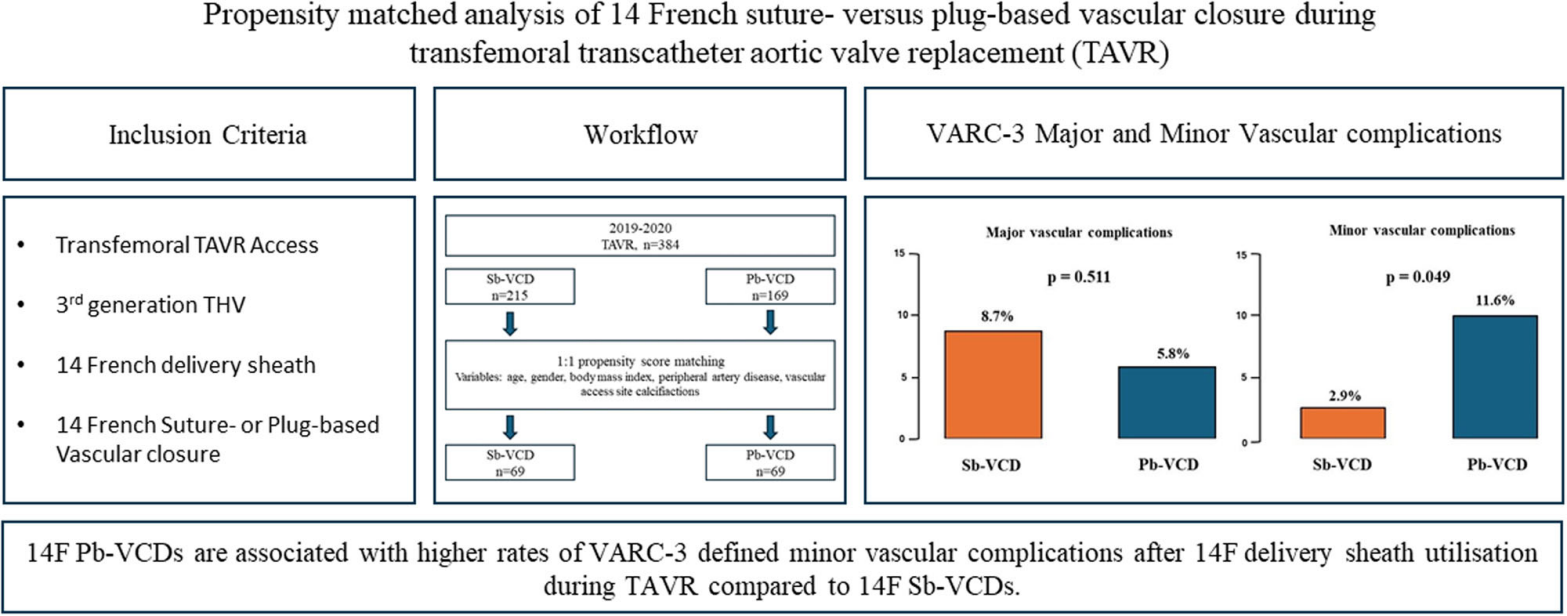
Summarizing illustration**.** Pb‐VCD, plug‐based vascular closure device; Sb‐VCD, suture‐based vascular closure device; TAVR, transcatheter aortic valve stenosis; THV, transcatheter heart valve; VARC‐3, Valve Academic Research Consortium–3 criteria; VCD, vascular closure device. [Color figure can be viewed at wileyonlinelibrary.com]

## 2 Methods

### 2.1 Study Design and Population

All patients who underwent percutaneous transfemoral transcatheter aortic valve replacement (TAVR) with a third‐generation THV (Medtronic Evolut R/PRO) and low‐profile 14 French (F) delivery sheath (Medtronic Sentrant, Medtronic EnVeo PRO) and received suture‐based (Sb‐) or plug‐based vascular closure devices (Pb‐VCD) were identified at two large German sites. Patients with non‐femoral access, implanted THV other than third‐generation Medtronic Evolut R/PRO, utilization of a VCD other than 14 F Perclose ProGlide (Abbott Vascular, Illinois, USA) or 14 F MANTA (Teleflex Inc, Morrisville, USA), or utilization of a large‐bore sheath other than 14 F were excluded. Indication for TAVR was judged by the local heart team including interventional cardiologists, trained cardiac surgeons, an anesthesiologist, and radiologists with 5 or more years of expertise in TAVR implantation and access site management. All procedures were performed by highly experienced operators in centers with an established multidisciplinary TAVR program. All operators were certified and experienced in the use of the applied VCD types.

The study was approved by the local ethics committees of the Heart Center of the Helios University Wuppertal/University Witten‐Herdecke and the German Heart Center Munich. All work involving human subjects was conducted in accordance with the World Medical Association Declaration of Helsinki. Further, all research was performed in accordance with relevant guidelines and regulations, and informed consent was obtained from all participants and/or their legal guardians.

### 2.2 Vascular Access and Closure

Procedural planning included contrast‐enhanced computerized tomography. Mean common femoral artery diameter, location of femoral artery bifurcation, and vessel tortuosity were assessed. Calcifications at the common femoral artery (CFA) were classified as mild, moderate, or severe if present [7, 8]. The feasibility of transfemoral TAVR in the presence of mild to severe femoral calcification at the vascular access site was evaluated by the local heart team. All TAVR operators have extended clinical experience in vascular access and closure including significant femoral vessel calcifications. Anticoagulation and pre‐existing antiplatelet therapy were not changed before the procedure. New oral anticoagulants (NOAC) were discontinued at least 24 h before the procedure. Intravenous unfractionated heparin was used to achieve a target activated clotting time of 250 s during the intervention.

Puncture of the CFA for TAVR access was guided by contralateral angiography. Ultrasound or different guidance techniques were not used. In none of the cases, ileo‐femoral access required pretreatment with percutaneous transluminal angioplasty or intravascular lithotripsy. The Medtronic THV was implanted with either 14 F Medtronic Sentrant or EnVeo PRO delivery systems. Since this study focused on 14 F devices, no Medtronic Evolut R/PRO 34 mm THV were included due to the requirement of a 16 F delivery sheath as a minimum. The company's guidelines required vessel predilatation with a 14 F sheath before insertion of the EnVeo PRO system. This initial sheath also facilitated potential aortic valve predilatation. If postdilatation of the THV was performed, a second exchange of large‐bore sheaths was required.

After removal of the large bore sheath over a guidewire, the access hole was closed using a dedicated VCD. In this study, the 14 F MANTA (Teleflex Inc, Morrisville, USA) Pb‐VCD system and the ProGlide (Abbott Vascular, Illinois, USA) Sb‐VCD system were used for vascular closure during TAVR. Handling details of the systems were described previously [4, 9, 10, 11, 12]. To reduce the activated clotting time after removal of large‐bore sheaths, protamine was administered at operators' discretion. If a residual bleeding was observed after VCD application, manual compression was applied for at least 3 min. Endovascular measures or surgical intervention were applied if further bleeding was observed or if bleeding was substantial. Adequacy of access site closure was ensured angiographically.

### 2.3 End Point

The primary endpoints of this trial were VARC‐3 defined major and minor vascular complications at the femoral access site during hospitalization for TAVR with a focus on bleeding and arterial obstruction due to stenosis or dissections. Secondary endpoints included length of hospital stay and intra‐hospital cardiovascular mortality.

### 2.4 Data Collection

Baseline characteristics, procedural details, intra‐hospital course, and adverse events were prospectively collected recording according to the VARC‐3 criteria recommendations [13]. For this study, all data were validated by reviewing operative reports, medical reports, and intra‐procedural angiographic studies. To determine femoral artery diameter and calcification, all available preoperative computerized tomographies underwent reassessment by the same examiner, unaware of the performed procedure and patient outcome. Vessel wall calcification at the access site between femoral bifurcation and cranial margin of the femoral head was graded as none, mild, moderate, or severe based on visual assessment. Adequate vessel diameter and absence of severe tortuosity of the iliac arteries to allow transfemoral access was confirmed.

### 2.5 Statistical Analysis

The data was analyzed using DataTab software (Datatab e.U., Graz, Austria) and SPSS Statistics 29 software (IBM, New York, USA). All tests are two‐sided and *p* < 0.05 is considered statistically significant. Tests for normal distribution were applied to metric variables. The Kolmogorov‐Smirnov test and the interpretation of the graphical Quantile−Quantile plot were performed. For normally distributed variables, the hypothesis test was carried out using the *T*‐test for independent samples. For non‐normally distributed variables, the Mann−Whitney *U*‐test was used. Normally distributed variables were documented as mean ± standard deviation. Non‐normally distributed variables were documented as median (Quartile 1−Quartile 3). If univariate nominal variables were compared with each other, the chi‐square test was performed. If the requirements for the chi‐square test were not met (< 5 expected observations), Fisher's exact test was performed.

A 1:1 nearest neighbor propensity score matching using SPSS Statistics 29 software was applied for baseline characteristics previously associated with vascular access site complications: age, gender, body mass index (BMI), peripheral vascular disease, and vascular calcifications at the access site.

## 3 Results

### 3.1 Baseline and Procedural Characteristics

**Figure 1:**
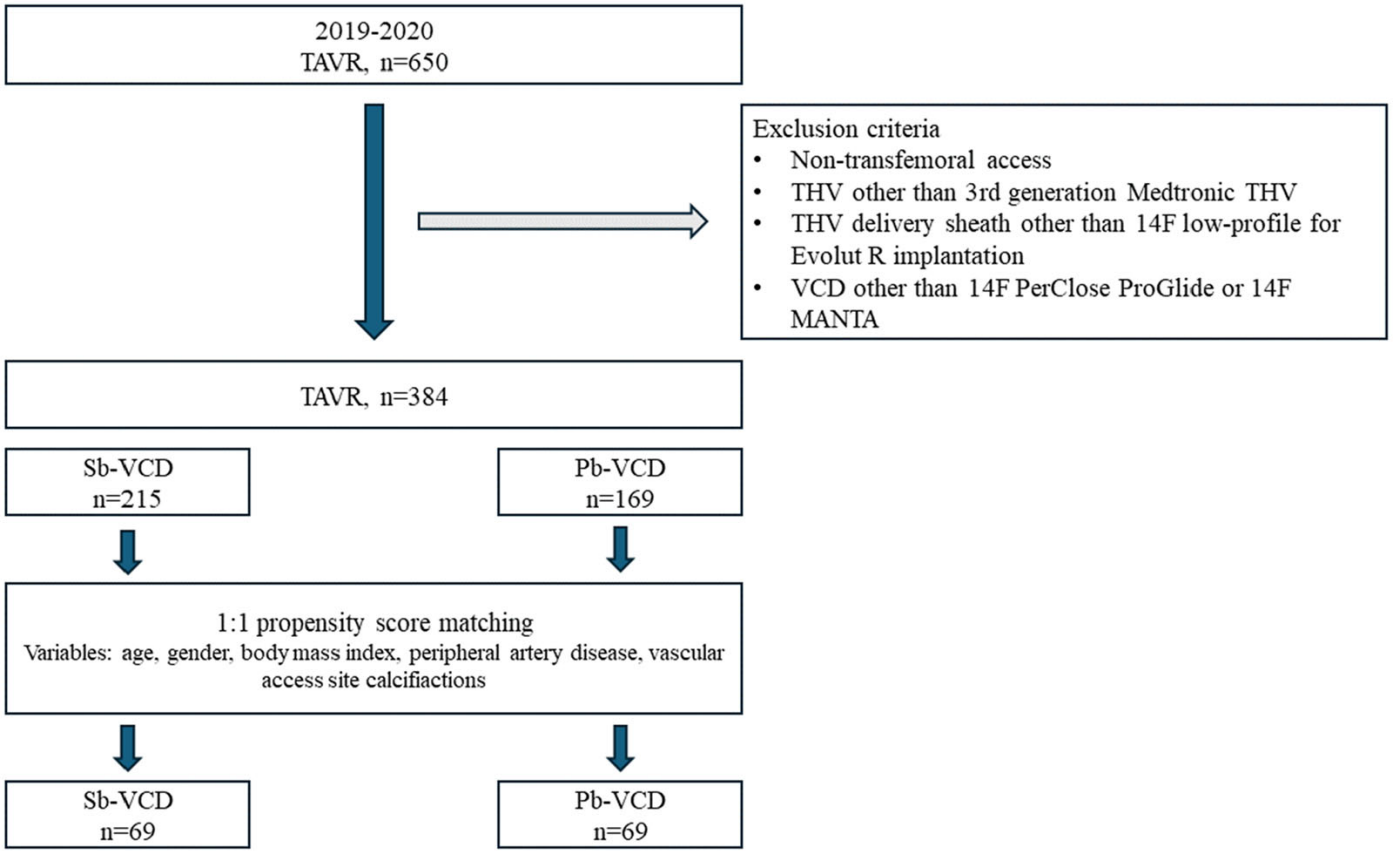
Study flow and variables for propensity score matching. Pb‐VCD, plug‐based vascular closure device; Sb‐VCD, suture‐based vascular closure device; TAVR, transcatheter aortic valve replacement; THV, transcatheter heart valve. [Color figure can be viewed at wileyonlinelibrary.com]

A total of 384 patients treated via transfemoral TAVR met the inclusion criteria for this study (Figure 1). The ProGlide (Abbott Vascular, Illinois, USA) Sb‐VCD was used in 215 patients, and 169 patients received the 14 F MANTA (Teleflex Inc, Morrisville, USA) Pb‐VCD following 14 F low‐profile delivery sheath use for THV implantation. Table 1 displays the baseline data of the Sb‐ and Pb‐VCD groups before and after propensity score matching. Differences in the groups before matching included age (79.5 ± 8.4 [Sb‐VCD] vs. 82.0 ± 5.9 [Pb‐VCD], *p* < 0.001), Euro Score 2 (3.7 [2.5−7.2] vs. 4.9 [3.3−7.4], *p* = 0.004), and ilio‐femoral tortuosity (22.8% vs. 75.1%, *p* < 0.001).

**Table 1:** Baseline characteristics.

	Entire cohort			Matched cohort		
*n*	Sb‐VCD 215	Pb‐VCD 169	*p* value	Sb‐VCD 69	Pb‐VCD 69	*p* value
Age (years)	79.5 ± 8.4	82.0 ± 5.9	**< 0.001**	81.6 (72.8−85.0)	82.8 (79.8−85.3)	0.085
Gender (female)	134 (62.3%)	116 (68.6%)	0.190	48 (69.6%)	50 (72.5%)	0.852
Body mass index (kg/m²)	25 (23−28)	26 (24−30)	0.196	25 (23−29)	26 (23−30)	0.282
Euroscore 2	3.7 (2.5−7.2)	4.9 (3.3−7.4)	**0.004**	4.9 (3.1−7.5)	4.1 (2.8−7.2)	0.825
Coronary artery disease	106 (49.3%)	87 (51.5%)	0.672	35 (50.7%)	31 (44.9%)	0.495
Coronary artery bypass grafting	15 (7.0%)	10 (5.9%)	0.676	5 (7.3%)	3 (4.4%)	0.218
Percutaneous coronary intervention	56 (26.1%)	51 (30.2%)	0.370	20 (29.0%)	20 (29.0%)	0.956
Peripheral artery disease	20 (9.3%)	18 (10.7%)	0.660	8 (11.6%)	6 (8.7%)	0.573
Atrial fibrillation	55 (25.6%)	65 (38.5%)	**< 0.007**	13 (18.8%)	28 (40.6%)	**0.005**
GFR (mL/min)	60.0 ± 21.9	51.5 ± 18.4	**< 0.001**	60.7 ± 22.1	54.5 ± 16.6	0.068
Anemia (Hb < 11 g/dL)	39 (18.1%)	65 (38.5%)	**< 0.001**	12 (17.4%)	36 (37.7%)	**0.008**
Access site CFA diameter (mm)	7.5 (7.0−8.4)	8.0 (7.0−9.0)	0.279	7.4 (6.9−8.0)	8.0 (7.0−9.0)	0.095
Ilio‐femoral tortuosity	49 (22.8%)	127 (75.1%)	**< 0.001**	18 (26.1%)	53 (76.8%)	**< 0.001**
CFA calcification ≥ moderate	60 (27.9%)	38 (22.5%)	0.226	20 (29.0%)	17 (24.6%)	0.564
LVEF (< 35%)	18 (8.4%)	3 (1.8%)	**0.005**	6 (8.7%)	1 (1.4%)	0.115

*Note:* Variables included in propensity score matching were age, gender, BMI, peripheral artery disease, and CFA calcifications. Variables highlighted in bold are significantly different between the groups.

Abbreviations: CFA, common femoral artery; GFR, glomerular filtration rate; LVEF, left ventricular ejection fraction; Pb‐VCD, plug‐based vascular closure system; Sb‐VCD, suture‐based vascular closure system.

### 3.2 Propensity Score Matching and Procedural Outcome

Propensity score matching was performed for variables previously associated with access site‐related vascular adverse events and included age, gender, peripheral artery disease, BMI, and vascular calcifications, resulting in 69 matched patient pairs without significant difference in previously matched parameters (Table 1). Parameters which were significantly different between the groups, but either not previously associated with vascular complications (anemia, GFR, atrial fibrillation) or potentially biased by non‐standardized definitions (vascular tortuosity) were not included as variables in propensity score matching.

### 3.3 Primary and Secondary Endpoints

**Figure 2:**
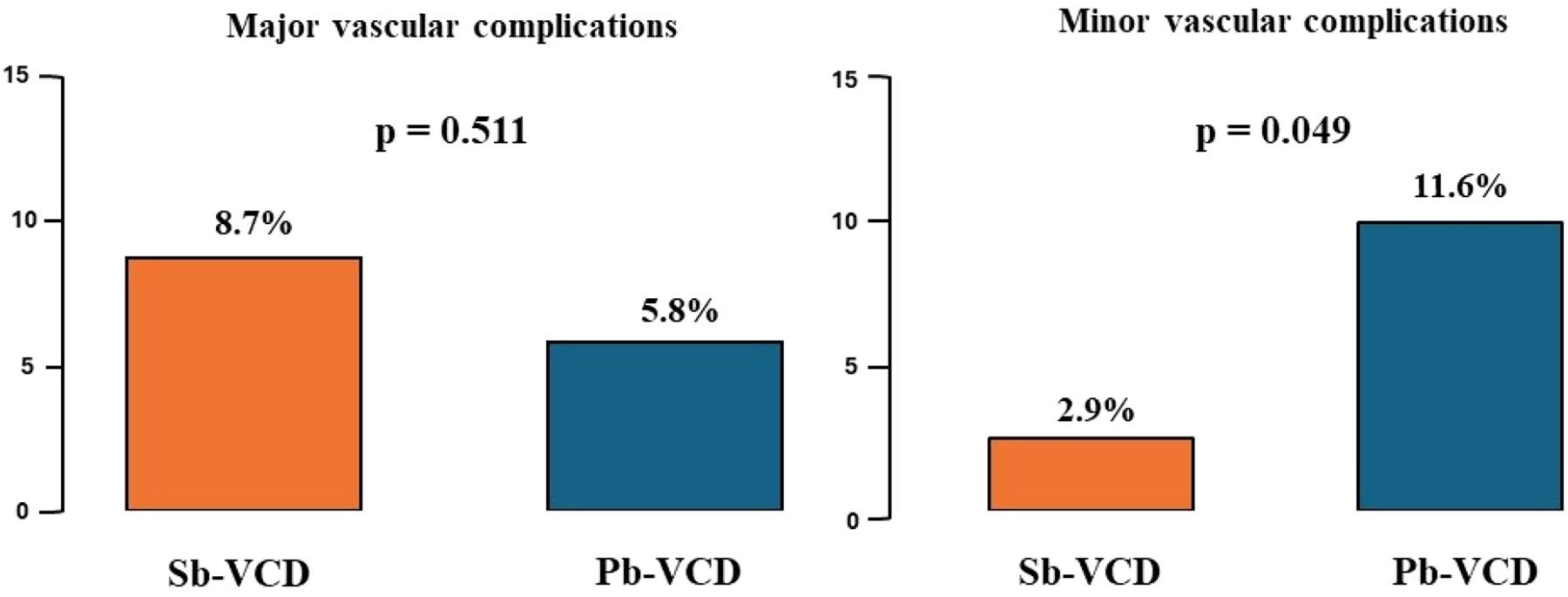
Major and minor Valve Academic Research Consortium‐3 (VARC‐3) defined vascular complications with the suture‐ (Sb‐VCD) or plug‐based vascular closure device (Pb‐VCD). [Color figure can be viewed at wileyonlinelibrary.com]

Minor VARC‐3 vascular complications occurred significantly more often in the Pb‐VCD group (2.9% vs. 11.6%, *p* = 0.049) and bleeding events showed a trend toward difference in favor of the Sb‐VCD group (5.8% vs. 14.5%, *p* = 0.091), not meeting the pre‐specified threshold for statistical significance but being the most common types of vascular complication observed (Table 2). Major VARC‐3 vascular complications were 8.7% in the Sb‐VCD group and 5.8% in the Pb‐VCD group (*p *= 0.511, Figure 2). Secondary endpoints consisting of length of stay in hospital (6 [IQR 4−7] vs. 6 [5−8] days, *p* = 0.270) and in‐hospital cardiovascular mortality (5.8% vs. 1.4%, *p* = 0.366) were comparable between both groups.

**Table 2:** Procedural data and outcome after transcatheter aortic valve replacement with plug‐based‐ (Pb‐) or suture‐based vascular closure systems (Sb‐VCD).

	Sb‐VCD	Pb‐VCD	*p* value
Patients, *n*	69	69	
Procedural data			
THV			
Evolut R/PRO	69 (100%)	69 (100%)	
THV size (mm)			
23	11 (15.9%)	2 (2.9%)	
26	45 (65.2%)	29 (42.0%)	
29	13 (18.8%)	38 (55.1%)	
34	0 (0.0%)	0 (0.0%)	
Procedural sheet			
Medtronic sentrant		69 (100%)	
EnVeo PRO	69 (100%)		
Access site CFA mean diameter (mm)	7.4 (6.9−8.0)	8.0 (7.0−9.0)	0.095
Fluoroscopy time (min)	13.0 (10.0−16.8)	9.0 (7.0−12.4)	**0.001**
Contrast agent (mL)	110 ± 25	197 ± 64	**< 0.001**
Outcome			
Major vascular complication	6 (8.7%)	4 (5.8%)	0.511
Minor vascular complication	2 (2.9%)	8 (11.6%)	**0.049**
Bleeding	4 (5.8%)	10 (14.5%)	0.091
Vessel stenosis, occlusion, dissection	4 (5.8%)	2 (2.9%)	0.681
Other	0 (0.0%)	0 (0.0%)	1.000
Length of hospital stay (days)	6 (4‐7)	6 (5‐8)	0.270
In‐hospital CV mortality	4 (5.8%)	1 (1.4%)	0.366

*Note:* Variables highlighted in bold are significantly different between the groups.

Abbreviations: CFA, common femoral artery; CV, cardiovascular; Pb‐VCD, plug‐based vascular closure system; Sb‐VCD, suture‐based vascular closure system; THV, transcatheter heart valve.

## 4 Discussion

The present study is a retrospective, propensity‐matched comparison that evaluated safety of 14 French (F) Pb‐VCD against the suture (Sb‐VCD) for percutaneous vascular access site closure after 14 F low‐profile third‐generation THV delivery sheath use in patients undergoing transfemoral transcatheter aortic valve replacement (TF‐TAVR). We aimed to elucidate a detailed understanding of the implications different 14 F VCDs have on vascular complications, compare our findings to previously published rates of vascular complications after larger 18 F VCD utilization during TAVR and to introduce first data comparing exclusively different 14 F vascular closure techniques after 14 F low‐profile third‐generation THV delivery sheath use. Our main findings are: (i) overall use of 14 F VCDs after low‐profile delivery sheath use is associated with < 9% of major vascular adverse events, and (ii) 14 F Pb‐VCDs did show significant higher rates of VARC‐3 minor vascular complications including occlusive or bleeding events in comparison to 14 F Sb‐VCDs despite the higher exchange rates of large bore sheaths for aortic valve pre‐ and postdilatation in Sb‐VCDs.

### 4.1 Risk Factors for Vascular Complications

Previous studies reported an association of age [14, 15], female gender [3, 16], BMI [17, 18, 19], peripheral artery disease [20, 21], and vascular calcifications [22, 23] with a higher risk for vascular complications in TAVR procedures. Further, exchange of a large‐bore sheath [2], as performed during EnVeo PRO delivery sheath use, and sheath size [5] were also identified as independent risk factors. Incidence of vascular complications was previously associated with training expertise regarding dedicated vascular closure devices of treating physicians [24]. Until now, a dedicated comparison between Sb‐ and Pb‐VCDs focussing exclusively on 14 F size and their influence on access‐related vascular complications has not been performed. In our cohort, we observed 8.7% of major and 2.9% of minor VARC‐3 defined vascular complications in the Sb‐VCD group and 5.8% and 11.6% (major/minor) in the Pb‐VCD group, respectively. Previous studies focused either on combined rates of vascular complications of 14F‐ together with 18 F Pb VCDs or included low number of patients, making interpretation of the available literature on 14 F Pb‐VCDs difficult [5, 25, 26]. Further, previous studies have a heterogenous subset of endpoint definitions including outcome measurements based on outdated VARC‐2 criteria [5, 27]. In our study, 14 F THV delivery sheaths used differed in the need for sheath exchange with further need for aortic valve pre‐ and postdilatation in the Sb‐VCD group. This serves as a potential factor which could have led to increased rates of vascular complications in this group.

### 4.2 Specifications of Suture‐ and Plug‐Based Vascular Closure Devices

The advent of catheter‐based endovascular therapies including TF‐TAVR for treatment of aortic stenosis has led to an increased need for dedicated percutaneous vascular access site closure devices. Closure of the arterial access site was initially achieved using surgical cut‐down and, later, using suture‐based VCDs [4]. Sb‐VCDs were recently challenged by the novel collagen‐based MANTA (Teleflex Inc, Morrisville, USA) Pb‐VCD. As previously described, application of the plug‐based system is intuitive with no need for a long‐lasting and complex learning curve [9, 25, 28]. Recently, 18 F Pb‐VCDs were associated with high rates of overall vascular complications of 19.4% in prospective, randomized trials [4]. Here, around 4% were VARC‐2 defined major vascular complications [4]. In comparison, previous trials including the 14 F MANTA (Teleflex Inc, Morrisville, USA) Pb‐VCD reported only 2% of major vascular complications and led to the hypothesis that the utilization of the 14 F device could reduce the incidence of vascular events [5]. Other studies that evaluated the 14 F device were able to report astonishing 0% of minor vascular complications according to old VARC‐2 definitions [25], However, in our recent evaluation, which is focussed solely on 14 F VCDs including the collagen‐based MANTA (Teleflex Inc, Morrisville, USA), significant higher rates need to be reported. Thus, VARC‐3 defined rates of vascular complications after smaller 14 F VCDs are comparable to previously published rates for larger 18 F devices.

In contrast, the ProGlide (Abbott Vascular, Illinois, USA) Sb‐VCD technique initially showed higher rates of vascular complications in non‐randomized studies [28, 29]. However, these results were not confirmed in randomized studies [4]. Similar to our results, the MASH trial (MANTA vs. Suture‐Based Vascular Closure After Transcatheter Aortic Valve Replacement) reported overall vascular complications of 10% in the MANTA (Teleflex Inc, Morrisville, USA) study arm [5]. Despite our nonsignificant findings regarding major VARC‐3 related vascular complications in this propensity matched comparison, the rate of minor vascular adverse events was able to reproduce the previous anticipated differences in the event rates between Sb‐ and Pb‐VCD favouring the suture‐based technique [4, 5].

## 5 Limitations

In our study, a comparingly low number of patient pairs resulted after propensity score matching. Thus, the incidence of bleeding events at the vascular access site could be statistically significantly different in cohorts including more patients. Further, even after propensity score matching, differences in baseline characteristics, including vascular tortuosity, did exist. Vascular access was not guided by ultrasound. However, all patients were treated by experienced interventionalists, thus a difference in treatment strategy as well as an impact of learning curve can be excluded. Further, all TAVR operators underwent TAVR device and VCD application training and certification including vascular access and closure strategies for calcified vessels. A potential bias of our study is the difference in outer diameter of the used sheaths. Larger, prospective clinical trials in an experienced TAVR center would be welcomed for further evaluation/comparison of 14 F versus 18 F Pb‐VCD during TAVR.

## 6 Conclusion

14 F Pb‐VCDs are associated with significantly higher rates of VARC‐3 defined minor vascular complications after 14 F delivery sheath utilization during TAVR, not leading to increased in‐hospital patients' mortality. Adequate vascular closure following transfemoral TAVR remains of high clinical significance and continuous efforts are needed to optimize vascular access and closure strategies.

## Ethics Statement

The study was approved by the local ethics committees of the Heart Center of the Helios University Wuppertal/University Witten‐Herdecke and the German Heart Center Munich. All work involving human subjects was conducted in accordance with the World Medical Association Declaration of Helsinki. Further, all research was performed in accordance with relevant guidelines and regulations, and informed consent was obtained from all participants and/or their legal guardians. Data can be obtained upon reasonable request.

## Conflicts of Interest

T.R. reports personal fees and others from Edwards, Novartis, Bristol Myers Squibb, Bayer, Daiichi Sankyo und AstraZeneca, and Pfizer outside of the submitted work. T.R. is a cofounder of Bimyo, a company focusing on the development of cardioprotective peptides. L.M. reports personal fees from Bayer, Alnylam, AstraZeneca, Bristol Myers Squibb, Pfizer, IFFM e.V., and from Bund der Niedergelassenen Kardiologen (BNK) outside of the submitted work. H.R. serves as a physician proctor for Abbott and Edwards Lifescience, a consultant for Medtronic, Abbott, and Edwards Lifescience, and a member of the Abbott advisory board. M.K. is a physician proctor and a member of the medical advisory board for JOMDD, a physician proctor for Peter Duschek, is a medical consultant for EVOTEC and Moderna and has received speakers' honoraria from Medtronic and Terumo. The other authors declare no conflicts of interest.

## Data Availability

The authors take responsibility for all aspects of the reliability and freedom from bias of the data presented and their discussed interpretation. Data is available upon reasonable request.
